# Drug-disease and drug-drug interactions: systematic examination of recommendations in 12 UK national clinical guidelines

**DOI:** 10.1136/bmj.h949

**Published:** 2015-03-11

**Authors:** Siobhan Dumbreck, Angela Flynn, Moray Nairn, Martin Wilson, Shaun Treweek, Stewart W Mercer, Phil Alderson, Alex Thompson, Katherine Payne, Bruce Guthrie

**Affiliations:** 1 University of Dundee, Mackenzie Building, Dundee DD2 4BF, UK; 2 Scottish Intercollegiate Guidelines Network, Healthcare Improvement Scotland, Edinburgh EH12 9EB, UK; 3 Raigmore Hospital, Inverness IV2 3UJ, UK; 4 University of Aberdeen, Health Services Research Unit, Foresterhill, Aberdeen AB25 2ZD, UK; 5 University of Glasgow, Institute of Health and Wellbeing, Glasgow G12 9LX, UK; 6 Centre for Clinical Practice, National Institute for Health and Care Excellence, Manchester M1 4BD; 7 Manchester Centre for Health Economics, Jean McFarlane Building, University of Manchester, Manchester M13 9PL, UK

## Abstract

**Objective** To identify the number of drug-disease and drug-drug interactions for exemplar index conditions within National Institute of Health and Care Excellence (NICE) clinical guidelines.

**Design** Systematic identification, quantification, and classification of potentially serious drug-disease and drug-drug interactions for drugs recommended by NICE clinical guidelines for type 2 diabetes, heart failure, and depression in relation to 11 other common conditions and drugs recommended by NICE guidelines for those conditions.

**Setting** NICE clinical guidelines for type 2 diabetes, heart failure, and depression

**Main outcome measures** Potentially serious drug-disease and drug-drug interactions.

**Results** Following recommendations for prescription in 12 national clinical guidelines would result in several potentially serious drug interactions. There were 32 potentially serious drug-disease interactions between drugs recommended in the guideline for type 2 diabetes and the 11 other conditions compared with six for drugs recommended in the guideline for depression and 10 for drugs recommended in the guideline for heart failure. Of these drug-disease interactions, 27 (84%) in the type 2 diabetes guideline and all of those in the two other guidelines were between the recommended drug and chronic kidney disease. More potentially serious drug-drug interactions were identified between drugs recommended by guidelines for each of the three index conditions and drugs recommended by the guidelines for the 11 other conditions: 133 drug-drug interactions for drugs recommended in the type 2 diabetes guideline, 89 for depression, and 111 for heart failure. Few of these drug-disease or drug-drug interactions were highlighted in the guidelines for the three index conditions.

**Conclusions** Drug-disease interactions were relatively uncommon with the exception of interactions when a patient also has chronic kidney disease. Guideline developers could consider a more systematic approach regarding the potential for drug-disease interactions, based on epidemiological knowledge of the comorbidities of people with the disease the guideline is focused on, and should particularly consider whether chronic kidney disease is common in the target population. In contrast, potentially serious drug-drug interactions between recommended drugs for different conditions were common. The extensive number of potentially serious interactions requires innovative interactive approaches to the production and dissemination of guidelines to allow clinicians and patients with multimorbidity to make informed decisions about drug selection.

## Introduction

Despite widespread multimorbidity, clinical guidelines are largely written as though patients have a single condition and the cumulative impact of treatment recommendations from multiple clinical guidelines is not generally considered.^1^ ^2^ In people with several conditions, simply application of recommendations from multiple single disease clinical guidelines can result in complex multiple drug regimens (polypharmacy) with the potential for implicitly harmful combinations of drugs.^3^ ^4^ ^5^ Clinical guidelines of course are not intended to be completely comprehensive guides to practice, in that clinicians are expected to use their judgment in deciding which treatments are appropriate in individual patients. There is, however, increasing recognition that clinical guidelines should better account for patients with multimorbidity.^2^ ^6^

Adverse drug events cause an estimated 6.5% of unplanned hospital admissions in the United Kingdom, accounting for 4% of hospital bed capacity.^7^ When an admission ends in death, these are predominately the result of bleeding or renal injury.^7^ While some adverse drug events are unpredictable (such as anaphylaxis from an unrecognised allergy), many others can be predicted and prevented, including drug-disease and drug-drug interactions.^8^ A considerable proportion of adverse drug events are caused by interactions between drugs.^9^ Systematic reviews have shown that electronic alerts and prompts can improve prescribing behaviour or reduce rates of error.^10^ Nevertheless, despite the increasing availability of computerised decision support, adverse drug events as a cause for seeking ambulatory care have increased, nearly doubling in the United States between 1995 and 2005, with increasing age and increasing polypharmacy being the predominant characteristics of patients associated with experiencing such an event.^4^ With an ageing population, and associated increasing multimorbidity, there is an increase in the potentially required number of drugs^11^ and so the potential for increased risk of drug interactions.^8^ ^12^ The American Geriatrics Society has identified the consideration of drug-disease and drug-drug interactions as a key element of optimal care for older adults with multimorbidity.^13^

We quantified how often the drugs recommended by three exemplar clinical guidelines from the National Institute of Health and Care Excellence (NICE) have drug-disease interactions in the presence of other commonly comorbid conditions or have potentially serious drug-drug interactions with drugs recommended by guidelines for these conditions.

## Methods

We selected three exemplar clinical guidelines produced by NICE, chosen because they were for common and important chronic physical and mental health conditions: heart failure,^14^ type 2 diabetes,^15^ and depression.^16^ Nine other NICE guidelines for potentially comorbid conditions were then selected. Guidelines were chosen on the basis of being a common and chronic condition; being recently published; including recommendations for the initiation of a drug treatment for a chronic condition, and being for conditions commonly comorbid with the three index conditions (fig 1). These nine conditions were atrial fibrillation,^17^ osteoarthritis,^18^ chronic obstructive pulmonary disease (COPD),^19^ hypertension,^20^ secondary prevention after myocardial infarction (post-myocardial infarction),^21^ dementia,^22^ rheumatoid arthritis,^23^ chronic kidney disease,^24^ and neuropathic pain.^25^

**Figure fig1:**
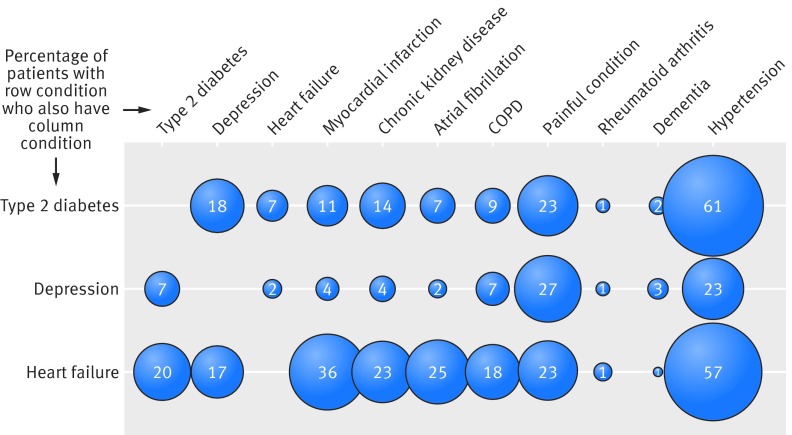
**Fig 1** Proportion of people with three index conditions who have each of other conditions. Morbidity data were not available for osteoarthritis or neuropathic pain; “painful condition” data shown are defined by receipt of four or more prescriptions for non-over the counter analgesics in previous 12 months

For each of the 12 guidelines, a panel of three clinicians (a general practitioner and two pharmacists) reviewed all recommendations made regarding the initiation of chronic drug treatments. We defined a drug as “first line” if it was recommended as a treatment for all or nearly all people with the condition, (for example angiotensin converting enzyme inhibitors for people with heart failure), whereas drugs that were recommended for only some patients with the condition under some circumstances were defined as “second line” (for example spironolactone for people with heart failure and high levels of symptoms despite first line treatment).

The expert panel then identified and classified drug-disease and drug-drug interactions and examined published guidelines to see if identified interactions were explicitly discussed. The *British National Formulary* (*BNF*) is the primary source used by UK clinicians to obtain information on drug-drug and drug-disease interactions.^26^ For each of the three exemplar index guidelines we systematically searched the *BNF* to identify drug-disease warnings for guideline recommended drugs, taking account of the predefined 11 conditions (the other two index conditions and the nine others). Drug-disease warnings were defined as being important if a disease was stated to be a contraindication in relation to all or most people with the condition, or if the *BNF* stated that drugs should be used only with caution accompanied by a clear statement to avoid in all or most people with the condition. For chronic kidney disease but not for other conditions, *BNF* warnings often recommended dose adjustment, and therefore this was additionally counted for this condition.

The *BNF* categorises drug-drug interactions by severity and defines potentially serious interactions as ones when “concomitant administration of the drugs involved should be avoided (or only undertaken with caution and appropriate monitoring).”^26^ Of note is that the “potentially serious” designation is not an indication of the likelihood of an interaction but of the seriousness of the potential harm if it occurs. The expert panel used the *BNF* to identify potentially serious interactions between drugs recommended by each of the three index guidelines and drugs recommended by any of the 12 guidelines (as two drugs recommended in the same guideline can interact). The expert panel then classified each identified drug-drug interaction in terms of the type of potential adverse effect caused. Disagreement between panel members was resolved by discussion to reach a consensus view. The potential harms of included interventions were then classified into risk of bleeding; central nervous system toxicity; cardiovascular adverse effect (including change in blood pressure or effect on heart rate or rhythm); and effect on renal function or serum potassium, or other. The “other” classification included risk associated with changes in concentration of drugs with a narrow therapeutic index such as lithium carbonate, digoxin, and theophylline.^27^ We chose these classification categories to reflect the types of adverse drug events associated with emergency admission to hospital.^7^

## Results

Table 1 shows the number of drugs or drug classes recommended as first line (for all or nearly all patients) and second line (for some patients under some circumstances) for each condition[Table tbl1] (details of the individual drugs are in appendix 1). There were 23 drugs recommended in the type 2 diabetes guideline (four first line), 13 drugs in the depression guideline (one first line), and 11 in the heart failure guideline (two first line).

**Table 1 tbl1:** Number of drugs recommended for each condition in each NICE guideline considered

Condition	Guideline No	Year published	No of drugs/drug classes recommended	
			First line	Second line
Type 2 diabetes	CG87	2009	4	19
Depression	CG90	2009	1	12
Heart failure	CG108	2010	2	9
Atrial fibrillation	CG36	2006	4	7
Dementia	CG42	2006	3	1
Secondary prevention post-MI	CG48	2007	4	13
Osteoarthritis	CG59	2008	2	5
Chronic kidney disease	CG73	2008	1	6
Rheumatoid arthritis	CG79	2009	9	9
Neuropathic pain	CG96	2010	2	5
Chronic obstructive pulmonary disease	CG101	2010	2	8
Hypertension	CG127	2011	4	3

NICE=National Institute of Health and Care Excellence; MI=myocardial infarction.

### Drug-disease interactions

Table 2 shows the number of times that a drug recommended for each of the three index conditions would be contraindicated or should be avoided in the presence of any of the other 11 conditions[Table tbl2]. Drug-disease interactions were not common, with the exception of those related to chronic kidney disease, which occurred with type 2 diabetes in particular. Chronic kidney disease was involved in 27 of the identified 32 drug-disease interactions for drugs recommended in the clinical guideline for type 2 diabetes and all of the six and 10 drug-disease interactions for the guidelines for depression and heart failure, respectively. But the guidelines for type 2 diabetes and heart failure each specifically discussed just one of these identified interactions. For type 2 diabetes this recommendation was regarding the need to avoid treatment with thiazolidinediones in people with comorbid heart failure. In heart failure, it was identified that amlodipine should be considered for the treatment of comorbid hypertension and/or angina in patients with heart failure, but verapamil, diltiazem, or short acting dihydropyridine agents should be avoided. The depression guideline did not discuss any of the identified drug-disease interactions.

**Table 2 tbl2:** Number of drug-disease interactions between drugs/drug classes recommended for each index condition and 11 other conditions in NICE guidelines. First line treatments are explicitly described as first line drugs or recommended for (almost) everyone with the condition; second line treatments (or other drugs) are explicitly described as second or third line drugs or recommended for only some subgroups or in some uncommon circumstances

Index conditions	CKD (dose change)	CKD (avoid)	Heart failure	Depression	Type 2 diabetes	Atrial fibrillation	Osteoarthritis	COPD	Hypertension	Post MI	Dementia	Rheumatoid arthritis	Neuropathic pain	Total
Type 2 diabetes*														
First line	3	2	0	0	—	0	0	0	0	0	0	0	0	5
Second line	11	11	5	0	—	0	0	0	0	0	0	0	0	27
**Depression†**														
First line	1	0	0	—	0	0	0	0	0	0	0	0	0	1
Second line	2	3	0	—	0	0	0	0	0	0	0	0	0	5
**Heart failure‡**														
First line	2	1	—	0	0	0	0	0	0	0	0	0	0	3
Second line	3	4	—	0	0	0	0	0	0	0	0	0	0	7

*CG87 type 2 diabetes: first line: metformin, sulphonylurea, angiotensin converting enzyme inhibitors (ACEI), simvastatin/atorvastatin. Second line/other: angiotensin II receptor antagonists (ARB) for hypertension, calcium channel blocker for hypertension, diuretic for hypertension, α blocker for hypertension, β blocker for hypertension, K-sparing diuretic for hypertension, other statins (not simvastatin/atorvastatin), fibrate, erythromycin, phosphodiesterase type-5 inhibitors (PPDE5 inhibitors), metoclopramide, ezetemibe, omega-3 fish oil, domperidone, DPP-4 inhibitor/gliptin, thiazolidinedione, GLP-1 mimetic (exenatide), acarbose, insulin.

†CG90 depression: first line: selective serotonin reuptake inhibitors. Second line: venlafaxine, mirtazepine, duloxetine, reboxetine, flupenthixol, tryptophan, mianserin, tricyclic antidepressant (TCA), monoamine oxidase inhibitor (MAOI), moclobemide, lithium, antipsychotic (aripiprazole, olanzapine, quetiapine, risperidone).

‡CG108 heart failure: first line: ACEI, β blocker licensed for heart failure. Second line/other: licensed aldosterone antagonist, digoxin, ARB, hydralazine, nitrate, loop diuretic, warfarin, amlodipine if comorbid hypertension/angina, aspirin if comorbid coronary heart disease.

### Drug-drug interactions

Potentially serious drug-drug interactions were common (fig 2). We identified 133 potentially serious interaction pairs for the type 2 diabetes guideline, of which 25 (19%) involved one of the four drugs recommended as first line treatments for all or nearly all patients (appendix 1). Nine of the recommended drugs for diabetes did not have any drug-drug interactions. For the depression guideline, we identified 89 potentially serious drug-drug interaction pairs, of which 19 (21%) involved the one drug class recommended as first line (selective serotonin reuptake inhibitor antidepressants). For heart failure, there were 111 potentially serious drug-drug interaction pairs identified, of which 21 (19%) involved the two drug classes recommended as first line.

**Figure fig2:**
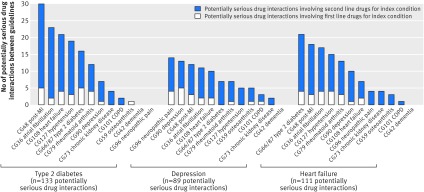
**Fig 2** Potentially serious drug-drug interactions between drugs recommended by clinical guidelines for three index conditions and drugs recommended by each of other 11 other guidelines

Figure 3 illustrates and table 3 summarises the types of harm expected from potentially serious drug-drug interactions by index condition (see appendix 2 for further detail). For type 2 diabetes, the most common category was cardiovascular related harm such as significant hypotension or bradycardia, followed by “other” (which includes increased lithium or digoxin concentrations causing risk of toxicity, and myopathy with statin treatment), and renal or serum potassium associated harms. For depression, the most commonly identified harm was risk of bleeding, particularly with selective serotonin reuptake inhibitors recommended as first line, followed by “other” harms (most commonly relating to lithium toxicity), and cardiovascular and central nervous system toxicity. Most of cardiovascular adverse effects in the depression guideline were related to increased risk of ventricular arrhythmias. The most common potentially serious drug interactions for the heart failure guideline were for bleeding events, but interactions causing severe hypotension or related to increased digoxin or lithium concentrations causing risk of toxicity were also cited.

**Figure fig3:**
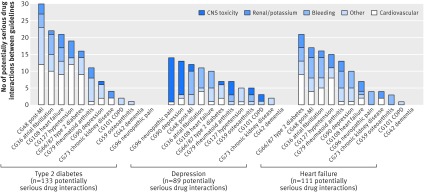
**Fig 3** Types of potentially serious harm from drug-drug interactions between drugs recommended by clinical guidelines for three index conditions and drugs recommended by each of other 11 other guidelines

**Table 3 tbl3:** Type of harm expected from potentially serious drug-drug interaction for each index condition

Index condition	Cardiovascular*	Bleeding	Renal/potassium	Central nervous system	Other†	Total
**Type 2 diabetes**						
First line recommended drug	3	3	2	0	12	20
Second line recommended drug	54	11	18	1	29	113
**Depression**						
First line recommended drug	1	9	0	7	2	19
Second line recommended drug	10	13	0	27	20	70
**Heart failure**						
First line recommended drug	15	0	4	0	2	21
Second line recommended drug	17	34	17	0	22	90

*Includes effects on heart rate or rhythm or effects on blood pressure.

†Includes myopathy with statin treatment, or clinically relevant altered plasma concentration (for example, of digoxin, lithium, ciclosporin, or theophylline), which might require dose alteration or closer monitoring.

A limited number of the identified drug-drug interactions were highlighted in the guidelines for the index condition. In the guideline for type 2 diabetes, only two interactions were mentioned: potassium sparing diuretics with angiotensin converting enzyme inhibitors; and potassium sparing diuretics with angiotensin receptor blockers. The depression guideline highlighted only the increased risk of bleeding with selective serotonin reuptake inhibitors plus non-steroidal anti-inflammatory drugs or aspirin. None of the recommendations in the heart failure guideline contained an explicit discussion of the 111 potentially serious drug-drug interactions identified.

## Discussion

### Principle findings

In this review of NICE clinical guidelines, potentially serious drug-drug interactions were relatively common among recommendations for each of three index conditions (type 2 diabetes, heart failure, and depression) and 11 other common conditions. In contrast, drug-disease interactions were relatively uncommon except in patients with comorbid chronic kidney disease. The types of harm potentially introduced by co-prescription of drugs varied by clinical guideline and were most commonly related to cardiovascular and “other” for drugs recommended for diabetes; bleeding and “other” for depression; and bleeding and cardiovascular for heart failure. Many guidelines suggest starting a drug treatment but currently rarely seem to consider drug-disease or drug-drug interactions in their recommendations.

### Comparison with other studies

Previous studies of the implications of following single disease guidelines in people with multimorbidity have usually considered single hypothetical patients with carefully selected multiple conditions; a scenario that is likely to overstate the scale of the problem.^5^ ^28^ Using US population survey data, Lorgunpai and colleagues found a much higher rate of drug-disease interactions (which they termed “therapeutic competition”), with a fifth of older American adults being prescribed drugs for one condition with the potential to worsen another.^29^ Their study, however, included interactions that did not reach our threshold of being recommended to avoid in all or most patients (for example, the use of β blockers for coronary heart disease in people with chronic obstructive pulmonary disease was common in their study but, although it carries some risk, is not stated as a contraindication or recommendation to avoid in the *BNF* because the benefits outweigh the harms in most patients).

### Strengths and limitations of study

One key potential limitation is the use of a selection of clinical guidelines as exemplar case studies, and some other guidelines do discuss interactions in more detail. For example, NICE has produced a guideline for depression in people with a chronic physical health problem^30^ that includes extensive discussion about drug interactions (although in a full guideline appendix, which will not be commonly read by clinicians) and a guideline on management of bipolar disorder^31^ that includes detailed recommendations about safe use of lithium. We would not, however, expect the pattern of findings to be substantially different for other guidelines that include a reasonable number of recommendations for chronic drug treatment. We excluded from our analysis any recommendations for starting drugs for acute conditions, but it should be noted that interactions with drugs like antibiotics and non-steroidal anti-inflammatory drugs (NSAIDs) used for short term intercurrent illness are common and important.^3^ The inclusion of additional guidelines would have further increased the number of potential interactions identified. Both of these exclusions imply that our findings are likely to be conservative.

We systematically examined recent national guidelines produced by NICE for important and common clinical conditions using data on interactions drawn from a single authoritative UK source. Definition of contraindications and potentially serious interactions was not straightforward, reflecting that the risk of such events is often poorly quantified and information sources vary in what is rated to be important.^32^ We used the *BNF* because it is the reference source used by most UK based clinicians. The *BNF* draws on data from a manufacturer’s summary of product characteristics (SPC), the medical literature, and expert opinion, but other reference sources might not be consistent with this, and databases of listed potentially serious drug interactions might have yielded different results. For example, a summary of product characteristics for amitriptyline from the online electronic medicines compendium of up to date, approved, and regulated prescribing information for licensed medicines^33^ includes history of myocardial infarction as a contraindication but the *BNF* 66th edition states only contraindication in the immediate recovery period after myocardial infarction.

### Conclusions and policy implications

We recommend that during the development of clinical guidelines the process should consider how to identify and more explicitly highlight the potential for interactions between recommended drugs and other conditions and other drugs that patients with the guideline condition are likely to have.^34^ ^35^ It is important to acknowledge that guideline developers have to maintain a balance between producing clear and relatively short recommendations and avoiding glossing over the complexity of the real world.^36^

For the conditions examined in our study, major drug-disease interactions were relatively rare with the exception of chronic kidney disease when interactions were more common. An implication is therefore that guideline developers should always explicitly decide whether chronic kidney disease is common enough in the real world population with the disease under consideration to require comment or modification of recommendations. For the three index conditions we examined, prevalence of comorbidity with chronic kidney disease was about 4% in patients with depression, 14% in patients with type 2 diabetes, and 23% in patients with heart failure. So the implication might be that guideline developers should consider chronic kidney disease with heart failure, possibly consider it with type 2 diabetes, and possibly not consider it with depression.

Potentially serious drug-drug interactions were much more common, but there are too many for all of them to be specifically mentioned by guidelines. From this perspective, we suggest that clinical guidelines produced and disseminated in a paper based format will only ever be able to adequately account for a minority of potential drug-drug interactions. Guideline developers should acknowledge potentially serious interactions and estimate their likely prevalence and severity. Prevalence will be determined both by whether the drug being recommended is first line (intended for all or nearly all people with the condition), by how commonly interacting drugs are used, which will depend on rates of comorbidity, and by how often the adverse drug event in question occurs. Of note is the requirement for detailed information about the real world population that the guideline is making recommendations for, which is currently much less commonly used in guideline development than trial data from narrowly selected populations. With the growth of large electronic primary care datasets, there is the option to define the population for which recommendations are being made and describe its demography, comorbidity, and current prescribing. As an example, there is a potentially serious interaction between statins recommended as first line treatment for patients with type 2 diabetes and ciclosporin recommended as second line for those with rheumatoid arthritis because of the risk of myopathy (and rhabdomyolysis). Given that only 1.4% of people with type 2 diabetes also have rheumatoid arthritis and that ciclosporin is recommended as second line only for those with rheumatoid arthritis, this will only ever be a rare drug-drug interaction and so is unlikely to reach the threshold for explicit consideration by a guideline development group. In contrast, co-prescription of selective serotonin reuptake inhibitors (recommended as first line treatment for patients with depression) and tramadol (recommended as second line for painful conditions) is likely to be common because tramadol is commonly used for pain in the UK and over 27% of people with depression also have painful conditions.^1^ ^37^ Although serotonin syndrome is potentially fatal, the risk of this condition seems to be low, but it is poorly quantified.^38^ The guideline development group will have to make a judgment as to whether they believe the interaction requires specific mention to inform clinicians and patients to be aware of the signs and symptoms of this syndrome should they occur. The key issue is that interactions and risks should be systematically assessed and explicit decisions made about whether they require discussion in a guideline, similar to the requirement for treatment benefits to be systematically and explicitly assessed. Details of expected harm from the identified potentially serious drug-drug interactions (such as those in appendix 2) should be considered to inform clinicians about alternative drug choices or to inform their discussions with individual patients.

Boxes 1 and 2 provide two illustrations of this. Box 1 describes a fairly simple set of drug-drug interactions in a woman with new diagnosis of depression after a myocardial infarction (in previous work, we found that 15% of people with a history of myocardial infarction have recent depression or are receiving antidepressants).^39^ The key issue is that selective serotonin reuptake inhibitors increase the risk of bleeding for someone already taking antiplatelet drugs (in this case dual treatment with aspirin and clopidogrel), and this issue is explicitly covered by NICE guidance.^30^

**Box 1—A new diagnosis of depression**

A 63 year old woman attends a GP appointment with her husband for follow-up of a new diagnosis of depression. She has had no benefit from low intensity psychosocial intervention and would like to discuss prescription for an antidepressant.

**Medical history**

Recent acute myocardial infarction (three months ago) and a history of hypertension. She struggles with increasing weight, which exacerbates flare up of pain from osteoarthritis in her knees. Current BMI is 32 and blood pressure 152/82 mm Hg at last check, eGFR >60 mL/min/1.73 m^2^.

**Current drug treatment**

Aspirin 75 mg daily, clopidogrel 75 mg daily, atorvastatin 40 mg daily, ramipril 5mg twice daily, atenolol 50 mg daily, paracetamol 500 mg as required. While discussing her drugs she explained that she also buys occasional ibuprofen when required for her knee pain because her prescribed paracetamol does not always relieve her pain.

**Considerations for depression management**

Selective serotonin reuptake inhibitors are recommended as the first line choice of antidepressant by the NICE guideline, but there are potentially serious drug interactions between selective serotonin reuptake inhibitors, aspirin, clopidogrel, and the ibuprofen the patient buys over the counter. Some alternative antidepressants including mirtazapine, duloxetine, and reboxetine do not have this interaction, although venlafaxine does.

**Outcome**

- After discussion with the patient a prescription for mirtazapine was started, and follow-up organised
- Regarding her knee pain and to reduce her risk of gastrointestinal bleeding, support was also organised for exercise and diet and co-codamol (paracetamol 500 mg/codeine 15 mg) was added as required for pain with caution regarding constipation and recommendation that she does not take ibuprofen
- She was offered a proton pump inhibitor for gastroprotection while taking dual antiplatelet therapy but declined as she was still coming to terms with having to take five drugs for cardiovascular secondary prevention.

Box 2 describes a more complex situation in a 72 year old man with a new diagnosis of type 2 diabetes who has pre-existing heart failure, atrial fibrillation, and chronic kidney disease (8.9% of people aged 70-79 with type 2 diabetes have heart failure, 9.4% have atrial fibrillation, and 10.4% have chronic kidney disease).^39^ Here treatment choices are more constrained in the face of multiple drug-drug and drug-disease interactions, and effective diabetes treatment might require more global treatment optimisation. In both cases, clinician judgment and patient involvement will be needed to make treatment decisions, but current guideline recommendations do not always support this process.

**Box 2—A new diagnosis of type 2 diabetes**

A 72 year old man attends with his wife for follow-up by his general practitioner regarding a new diagnosis of type 2 diabetes. He has not managed to control HbA_1C_ with a trial of lifestyle interventions, despite strictly adhering to diet. Examination showed mild ankle oedema. Short of breath after about half flight stairs. Results show HbA1c 60 mmol/mol (normal range 31-44 mmol/mol, equivalent to HbaA1c 8% normal range 5.0-6.2%) BMI 24, blood pressure 102/68 mm Hg, pulse 50 beats/min.

**Medical history**

He has longstanding hypertension and atrial fibrillation. He was investigated for increased shortness of breath two years ago and found to have moderate left ventricular systolic dysfunction. He has chronic kidney disease with a most recent eGFR of 37 mL/min/1.73 m^2^ (previously fluctuating between 34 and 39) and creatinine 129 µmol/L (normal range 50-120 µmol/L).

**Current drug treatment**

Ramipril 2.5 mg daily, spironolactone 25 mg daily, bisoprolol 5 mg daily, simvastatin 40 mg daily, amlodopine 5 mg daily, furosemide 40 mg daily, warfarin as per international normalised ratio.

**Considerations for management of type 2 diabetes**

NICE guidelines recommend several lifestyle and pharmacological interventions:

- Continuation of lifestyle measures for management of diabetes
- Management of cardiovascular risk
- Pharmacological interventions are already in place to manage his lipids and blood pressure, and he is a non-smoker
- Management of hyperglycaemia:
  Metformin can be used with caution in renal function, review dose if eGFR <45 mL/min/1.73 m^2^, and avoid if eGFR <30 mL/min/1.73 m^2^
  Option to start metformin with close monitoring of renal function
  Sulphonylureas have a potentially serious drug interaction with warfarin, causing changes to anticoagulant effect and enhanced hypoglycaemic effect
  Option to start sulphonylurea and close monitoring of international normalised ratio or
  Option to start sulphonylurea and change warfarin to a newer anticoagulant such as rivaroxaban
  Pioglitazone is contraindicated with heart failure.
- Also potentially serious interaction between amlodipine and simvastatin with increased risk of myopathy (the amlodipine might also be contributing to the ankle oedema)

**Outcome**

- Discussed diabetic control in context of other conditions. Patient chose to start metformin to help manage hyperglycaemia
- He was concerned about instability in his international normalised ratio. He would also like to consider change from warfarin to rivaroxaban at some point in the future but is reluctant to start too many new drugs at once
- Stop amlodipine (as it might be contributing to ankle swelling and his blood pressure is currently well controlled) and monitor blood pressure. Also the drug interaction would require a review of the statin therapy
- Blood pressure might recover enough to allow increased dose of ramipril, which will help nephropathy and heart failure (renal function permitting)

One of the challenges for guideline developers is that the actual harms of many drug-drug and drug-disease interactions are poorly quantified, partly reflecting that whereas clinical trials produce high quality evidence about benefit, they are poorly suited to estimating harms, particularly in real world populations in which people are typically older, frailer, have more multiple illnesses. and prescribed more drugs for other conditions than trial populations.^40^ Research is needed to more systematically quantify these harms because understanding when harms outweigh benefits is critical for rational treatment decisions, and better understandings of harm and the implications for the extrapolation of trial findings to real world populations will need to be systematically incorporated into existing guideline development frameworks like GRADE.^41^ Paper based single disease guidelines are intrinsically limited by being hard to integrate for people with multiple conditions and by being unable, for reasons of length and usability, to document all possible interactions. In principle, guidelines embedded in electronic medical records that integrate recommendations for all the conditions an individual has could deal with the problem we identified, including the difficulty of accounting for high levels of complexity such as the patient described in box 2, but the best design and effectiveness of such guidelines requires more research.^42^

**What is already known on this topic**

There is increasing recognition that clinical guidelines should better account for patients with multimorbidity
Many guidelines recommend drug treatments, but current guidelines rarely consider drug-disease or drug-drug interactions in these recommendations

**What this study adds**

For the 12 guidelines examined, drug-disease interactions were relatively uncommon, with the exception of interactions when an individual has comorbid chronic kidney disease
Potentially serious drug-drug interactions were common, although the harm caused will depend on both how commonly different conditions are comorbid and the prevalence and severity of the harm caused by the interaction
Guideline developers need to more explicitly account for drug-disease and drug-drug interactions in people with multimorbidity and should use epidemiological evidence to identify when interactions are likely to be common and serious enough to require specific mention in a guideline.
Guideline developers are currently limited by the use of paper based guidelines. Adaptive electronic based guidelines that allow interactive searching for specific conditions are a potential way forward to account for multimorbidity in guideline recommendations

## Notes

We acknowledge the work of the project reference team for the Better Guidelines for Better Care study, including Ian Lewin, Alison Allen, Graham Bell, Mark Davis, Roger Gadsby, John Hindle, Claudette Allerdyce, Sarah Davis, Carolyn Chew-Graham, Bhash Naidoo, Suzanne Lucas, Peter Rice, and Hugh McIntyre.

Ethical approval: Not required.

Data sharing: No additional data available.

Transparency: SD (the lead author and the manuscript’s guarantor) affirms that the manuscript is an honest, accurate, and transparent account of the study being reported; that no important aspects of the study have been omitted; and that any discrepancies from the study as planned (and, if relevant, registered) have been explained.

## Notes

Cite this as: *BMJ* 2015;350:h949

## References

[1] Barnett K, Mercer S, Norbury M, Watt G, Wyke S, Guthrie B. Epidemiology of multimorbidity and implications for healthcare, research, and medical education: a cross-sectional study. Lancet 2012;380:37-43 22579043 10.1016/S0140-6736(12)60240-2

[2] Guthrie B, Payne K, Alderson P, McMurdo M, Mercer S. Adapting clinical guidelines to take account of multimorbidity. BMJ 2012;345:e6341. 23036829 10.1136/bmj.e6341

[3] Guthrie B, McCowan C, Davey P, Simpson CR, Dreischulte T, Barnett K. High risk prescribing in primary care patients particularly vulnerable to adverse drug events: cross sectional population database analysis in Scottish general practice. BMJ 2011;342:d3514. 21693525 10.1136/bmj.d3514

[4] Bourgeois FT, Shannon MW, Valim C, Mandl KD. Adverse drug events in the outpatient setting: an 11-year national analysis. Pharmacoepidemiol Drug Safety 2010;19:901-10 10.1002/pds.1984 PMC2932855 20623513

[5] Boyd C, Darer J, Boult C, Fried L, Boult L, Wu A. Clinical practice guidelines and quality of care for older patients with multiple comorbid diseases implications for pay for performance. JAMA 2005;294:716-24 16091574 10.1001/jama.294.6.716

[6] House of Commons Health Committee. Managing the care of people with long-term conditions. Stationery Office, July 2014:222 (HC401). www.publications.parliament.uk/pa/cm201415/cmselect/cmhealth/401/401.pdf.

[7] Pirmohamed M, James S, Meakin S, Green C, Scott AK, Walley T, et al. Adverse drug reactions as cause of admission to hospital: prospective analysis of 18 820 patients. BMJ 2004;329:15-9. 15231615 10.1136/bmj.329.7456.15 PMC443443

[8] Calderon-Larranaga A, Poblador-Plou B, Gonzalez-Rubio F, Gimeno-Feliu LA, Abad-Diez JM, Prados-Torres A. Multimorbidity, polypharmacy, referrals, and adverse drug events are we ng things well? Br J Gen Prac 2012;62:e821-6 10.3399/bjgp12X659295 PMC3505415 23211262

[9] Marengoni A, Pasina L, Concoreggi C, Martini G, Brognoli F, Nobili A, et al. Understanding adverse drug reactions in older adults through drug-drug interactions. Eur J Intern Med 2014;25:843-6. 25312593 10.1016/j.ejim.2014.10.001

[10] Schedlbauer A, Prasad V, Mulvaney C, Phansalkar S, Stanton W, Bates DW, et al. What evidence supports the use of computerized alerts and prompts to improve clinicians’ prescribing behavior? J Am Med Inform Assoc 2009;16:531-8. 19390110 10.1197/jamia.M2910 PMC2705257

[11] Hovstadius B, Hovstadius K, Astrand B, Petersson G. Increasing polypharmacy—an individual-based study of the Swedish population 2005-2008. BMC Clin Pharmacol 2010;10:16 21122160 10.1186/1472-6904-10-16 PMC3014875

[12] Zhang M, Holman CD, Price SD, Sanfilippo FM, Preen DB, Bulsara MK. Comorbidity and repeat admission to hospital for adverse drug reactions in older adults: retrospective cohort study. BMJ 2009;338:a2752. 19129307 10.1136/bmj.a2752 PMC2615549

[13] American Geriatrics Society Expert Panel on the Care of Older Adults with Multimorbidity. Guiding principles for the care of older adults with multimorbidity: an approach for clinicians. J Am Geriatr Soc 2012;60:e1-25. 22994865 10.1111/j.1532-5415.2012.04188.x PMC4450364

[14] Chronic heart failure. Management of chronic heart failure in adults in primary and secondary care (NICE Clinical Guideline 108). National Institute for Health and Care Excellence, 2010.

[15] The management of type 2 diabetes (NICE Clinical Guideline 87). National Institute for Health and Care Excellence, 2010.

[16] The treatment and management of depression in adults (NICE Clinical Guideline 90). National Institute for Health and Care Excellence, 2009.

[17] The management of atrial fibrillation (NICE Clinical Guideline 36). National Institute for Health and Care Excellence, 2006.

[18] The care and management of osteoarthritis in adults (NICE Clinical Guideline 59). National Institute for Health and Care Excellence, 2008.

[19] Management of chronic obstructive pulmonary disease in adults in primary and secondary care (partial update) (NICE Clinical Guideline 101). National Institute for Health and Care Excellence, 2010.

[20] Clinical management of primary hypertension in adults (NICE Clinical Guideline 127). National Institute for Health and Care Excellence, 2011.

[21] Secondary prevention in primary and secondary care for patients following a myocardial infarction (NICE Clinical Guideline 48). National Institute for Health and Care Excellence, 2007.

[22] Supporting people with dementia and their carers in health and social care (NICE Clinical Guideline 42). National Institute for Health and Care Excellence, 2006 (amended 2011).

[23] The management of rheumatoid arthritis in adults (NICE Clinical Guideline 79). National Institute for Health and Care Excellence, 2009.

[24] Early identification and management of chronic kidney disease in adults in primary and secondary care (NICE Clinical Guideline 73). National Institute for Health and Care Excellence, 2008. 25340245

[25] The pharmacological management of neuropathic pain in adults in non-specialist settings (NICE Clinical Guideline 96). National Institute for Health and Care Excellence, 2010. 22319807

[26] British National Formulary. 66th ed. British Medical Association and Royal Pharmaceutical Society of Great Britain, 2013.

[27] Kesselheim AS, Misono AS, Lee JL, Stedman MR, Brookhart MA, Choudhry NK, et al. Clinical equivalence of generic and brand-name drugs used in cardiovascular disease: a systematic review and meta-analysis. JAMA 2008;300:2514-26. 19050195 10.1001/jama.2008.758 PMC2713758

[28] Hughes LD, McMurdo ME, Guthrie B. Guidelines for people not for diseases: the challenges of applying UK clinical guidelines to people with multimorbidity. Age Ageing 2013;42:62-9 22910303 10.1093/ageing/afs100

[29] Lorgunpai SJ, Grammas M, Lee DS, McAvay G, Charpentier P, Tinetti ME. Potential therapeutic competition in community-living older adults in the US: use of medications that may adversely affect a coexisting condition. PloS One 2014;9:e89447 24586786 10.1371/journal.pone.0089447 PMC3934884

[30] Depression in adults with a chronic physical health problem (NICE Clinical Guideline 91): Treatment and management. National Institute for Health and Care Excellence, 2009. 31886974

[31] Bipolar disorder: The management of bipolar disorder in adults, children and adolescents, in primary and secondary care (NICE Clinical Guideline 38). National Institute for Health and Care Excellence, 2006.

[32] Tan K, Petrie KJ, Faasse K, Bolland MJ, Grey A. Unhelpful information about adverse drug reactions. BMJ 2014;349:g5019 25115975 10.1136/bmj.g5019

[33] Amitriptyline BP tablets 10mg. Summary of product characteristics. Datapharm Communications, Actavis UK, March 2011.

[34] Uhlig K, Leff B, Kent D, Dy S, Brunnhuber K, Burgers JS, et al. A framework for crafting clinical practice guidelines that are relevant to the care and management of people with multimorbidity. J Gen Intern Med 2014;29:670-9 24442332 10.1007/s11606-013-2659-y PMC3965742

[35] Boyd C, Kent D. Evidence-based medicine and the hard problem of multimorbidity. J Gen Intern Med 2014;29:552-3 24442331 10.1007/s11606-013-2658-z PMC3965745

[36] Pronovost PJ. Enhancing physicians’ use of clinical guidelines. JAMA 2013;310:2501-2 24310916 10.1001/jama.2013.281334

[37] Ruscitto A, Smith B, Guthrie B. Changes in opioid and other analgesic use 1995-2010: repeated cross-sectional analysis of dispensed prescribing for a large geographical population in Scotland. Eur J Pain 2014;19:59-66. 24807782 10.1002/ejp.520

[38] Boyer EW, Shannon M. The serotonin syndrome. N Engl J Med 2005;352:1112-20 15784664 10.1056/NEJMra041867

[39] Barnett K, Mercer SW, Norbury M, Watt G, Wyke S, Guthrie B. Epidemiology of multimorbidity and implications for health care, research, and medical education: a cross-sectional study. Lancet 2012;380:37-43 22579043 10.1016/S0140-6736(12)60240-2

[40] Van Spall H, Toren A, Kiss A, Fowler R. Eligibility criteria of randomized controlled trials published in high-impact general medical journals a systematic sampling review. JAMA 2007;297:8 10.1001/jama.297.11.1233 17374817

[41] Andrews JC, Schünemann HJ, Oxman AD, Pottie K, Meerpohl JJ, Coello PA, et al. GRADE guidelines: 15. Going from evidence to recommendation—determinants of a recommendation’s direction and strength. J Clin Epidemiol 2013;66:726-35 23570745 10.1016/j.jclinepi.2013.02.003

[42] Vandvik PO, Brandt L, Alonso-Coello P, Treweek S, Akl EA, Kristiansen A, et al. Creating clinical practice guidelines we can trust, use, and share a new era is imminent. CHEST 2013;144:381-9. 23918106 10.1378/chest.13-0746

