# Supplementary material for: Drug-disease and drug-drug interactions: systematic examination of recommendations in 12 UK national clinical guidelines
Source: BMJ. 2015 Mar 11;350:h949. doi: 10.1136/bmj.h949 (PMC4356453; doi:10.1136/bmj.h949)
Supplement: Supplementary file 1 [file dums022733.ww1_default.pdf]

Appendix 1

First and second line drugs recommended in 12 selected NICE Clinical Guidelines.

Definitions

First line drug = explicitly stated to be a first-line drug, or recommended for (almost) everyone with the condition.
Second-line/other drugs = explicitly stated to be a second or third line drug, or recommended for only some subgroups or in some not very common circumstances

Identified first and second line drugs

1.- CG87 Type 2 Diabetes
First line: Metformin, sulphonylurea, angiotensin-converting enzyme inhibitors (ACEI), simvastatin/atorvastatin.
Second line/other: Angiotensin-II receptor antagonists (ARB) for hypertension, calcium channel blocker for hypertension, diuretic for hypertension, alpha blocker for hypertension, beta-blocker for hypertension, K-sparing diuretic for hypertension, other statins (not simvastatin/atorvastatin), fibrate, erythromycin, phosphodiesterase type-5 inhibitors (PPDE5 inhibitors), metoclopramide, ezetemibe, omega-3 fish oil, domperidone, DPP-4 inhibitor/gliptin, thiazolidinedione, GLP-1 mimetic (exenatide), acarbose, insulin.

2. CG90 Depression
First line: Selective serotonin reuptake inhibitors (SSRI).
Second line: Venlafaxine, mirtazepine, duloxetine, reboxetine, flupenthixol, tryptophan, mianserin, tricyclic antidepressant (TCA), monoamine oxidase inhibitor (MAOI), moclobemide, lithium, antipsychotic (aripiprazole, olanzapine, quetiapine, risperidone)

3. CG108 Heart Failure
First line: ACEI, beta blocker licensed for heart failure
Second line/other: Licenced aldosterone antagonist, digoxin, ARB, hydralazine, nitrate, loop diuretic, warfarin, amlodipine if comorbid hypertension/angina, aspirin if comorbid Coronary Heart Disease

4. CG36 Atrial Fibrillation
First line: Warfarin, aspirin, standard beta blocker, rate limiting calcium channel blocker.
Second line/other: Phenindione, dabigatran (rivaroxaban, apixaban), digoxin, sotalol, amiodarone, class 1c flecainide, class 1c propafenone

5. CG42 Dementia
First line: Donepezil, galantamine, rivastigmine.
Second line/other: memantine.

6. CG 48 Secondary Prevention Post–MI
First line: ACEI, aspirin. beta-blocker, statin.

Second line/other: ARB, clopidogrel, warfarin, diltiazem if beta-blocker contraindicated/not tolerated or hypertension/angina and not heart failure, verapamil if beta-blocker contraindicated/not tolerated or hypertension/angina and not heart failure, amlodipine for hypertension/angina and heart failure, proton pump inhibitor (PPI), aldosterone antagonist licensed post-MI, fibrates if intolerant of statin, omega 3 fatty acids, PPDE5 inhibitors, nicotine replacement therapy (NRT), bupropion.

7. CG59 Osteoarthritis
First line: Paracetamol, topical NSAID.
Second line/other: Oral non-selective NSAID, cyclo-oxygenase-2 selective inhibitors, PPI if on NSAID, opioids (codeine/dihydrocodeine), topical capsaicin.

8. CG73 CKD
First line: ACEI
Second line/other: Statins, antiplatelets, antihypertensives, ARB, vitamin D supplements, bisphosphonate.

9. CG79 Rheumatoid Arthritis
First line: Methotrexate, sodium aurothiomalate, penicillamine, hydroxychloroquine, chloroquine, azathioprine, ciclosporin, leflunomide, sulfasalazine
Second line/other: Adalimumab, etanercept, infliximab, paracetamol, codeine, dihydrocodeine, NSAIDs, Cyclo-oxygenase-2 selective inhibitors, PPI

10. CG96 Neuropathic Pain
First line: Amitriptyline, pregabalin.
Second line/other: Imipramine, nortriptyline, topical lidocaine, tramadol, duloxetine for painful diabetic neuropathy.

11. CG101 COPD
First line: Short-acting beta2 agonist (SABA), short-acting muscarinic antagonist (SAMA).
Second line/other: Long-acting muscarinic antagonist (LAMA), long- acting beta2 agonist (LABA), inhaled corticosteroid, theophylline, mucolytic, NRT, varenicline, bupropion.(Excluded: palliative, oxygen, long term steroids, osteoporosis)

12: CG127 Hypertension
First line: ACEI, ARB, calcium channel blocker, thiazide like diuretic.
Second line/other: Spironolactone, beta blocker, alpha blocker.
