# Supplementary material for: Drug-disease and drug-drug interactions: systematic examination of recommendations in 12 UK national clinical guidelines
Source: BMJ. 2015 Mar 11;350:h949. doi: 10.1136/bmj.h949 (PMC4356453; doi:10.1136/bmj.h949)
Supplement: Supplementary file 2 [file dums022733.ww2_default.pdf]

Appendix 2

Details of expected harm from the identified potentially serious drug-drug interactions for each of the three index conditions.

Index condition **Interacting drugs recommended by any of the 12 guidelines**

**Type 2 Diabetes**	ACEI	ARB	K sparing diuretic	Spironolactone	Aldosterone antagonist	Alpha blocker	Thiazide	Loop diuretic	Nitrate	MAOI	Sodium Aurothiomalate	Beta-blocker	Ca channel blocker	Amiodarone	Sotalol	Flecainide	Domperidone	Reboxetine	Ciclosporin	Lithium	Digoxin	Theophylline	Antipsychotic	Clopidogrel	Dabigatran	Phenindione	Statin	NSAIDs	Warfarin
**Metformin**																													
**Sulfonylurea**																												P	P
**ACE Inhibitor**			K+	K+	K+		BP-	BP-			BP-								K+	P									
**Simvastatin / Atorvastatin**													M	M					M										
ARB for hypertension			K+	K+	K+		BP-	BP-											K+	P									
Ca channel blocker for hypertension						BP-						HR-		HR-	HR-	HR-			P		P	P			B		M		
Diuretic for hypertension	BP-	BP-				BP-									K-	K-				P	K-								
Alpha blocker for hypertension			BP-	BP-			BP-	BP-		BP-		BP-	BP-		BP-														
Beta blocker for hypertension						BP-							HR-	HR-		HR-			P										
K-sparing diuretic for hypertension	K+	K+																		P									
Other statins																			M							B			P
Fibrate																										B	M		P
Erythromycin													P				HR+	P	P			P	P	B			M		B
PPDE5 inhibitor						BP-			BP-																				
Metoclopramide																			P										
Domperidone																													
Ezetemibe																													
Omega-3 fish oil																													
DPP-4 inhibitor/gliptin																													
Thiazolidinedione																													
GLP-1 mimetic (exenatide)																													
Acarbose																													
Insulin																													

Index condition	Interacting drugs recommended by any of the 12 guidelines																						
**Depression**	Aspirin	Warfarin	NSAID	Dabigatran	Clopidogrel	Alpha blocker	Theophylline	ARB	Ace Inhibitor	Diuretics	Lithium	Bupropion	codeine/dihydrocodeine	MAOI	Moclobemide	Tramadol	TCA	Antipsychotic	Erythromycin	Amiodarone	Flecainide	Propafenone	Sotalol
**SSRI**	B	B	B	B	B		P				P			CNS	CNS	CNS	VA	VA					
Venlafaxine	B	B	B	B										CNS	CNS			VA					
Mirtazapine														CNS	CNS								
Duloxetine				B										CNS	CNS								
Reboxetine														CNS									
Flupentixol																							
Tryptophan														CNS									
TCA		B												CNS	CNS	CNS		VA		VA	VA	VA	VA
MAOI						BP						CNS	CNS	CNS	CNS	CNS	CNS	CNS					
Moclobemide					B							CNS	CNS	CNS			CNS						
Lithium			P					P	P	P										VA			
Antipsychotic (aripiprazole, olanzapine, quetiapine, risperidone)										VA							VA	VA	VA	VA	VA		VA

Index condition **Interacting drugs recommended by any of the 12 guidelines**

**HEART FAILURE**	Alpha blocker	Sodium Aurothiomalate	PPDE5 inhibitor	Diuretic for BP	ACE Inhibitor	Angiotensin-II receptor antagonist	Digoxin	K+sparing diuretic	Flecainide	Sotalol	Amisulpride / Pimozide	Amiodarone	Propafenone	Ciclosporin	Verapamil	Diltiazem / Nifedipine	Nicardipine	Hydroxychloroquine Chloroquine	Lithium	Theophylline	Methotrexate	Phenindione	NSAIDs / COX2	SSRI	Venlafaxine	Erythromycin	Simvastatin	Sulphonylurea	Aspirin<br>Azathioprine<br>Clopidogrel<br>Dabigatran<br>Esomeprazole<br>Fibrates<br>Fluvastatin<br>Omeprazole<br>Rosuvastatin<br>TCA<br>Tramadol
Licenced Betablocker	BP-								HR-			HR-	HR-	P	BP-	HR-													
ACE I		BP-		BP-				HK						HK					P										
Aldosterone antagonist					HK	HK	P	HK						HK					P										
Digoxin				LK								P	P	P	P	P	P	P											
Angiotensin-II receptor antagonist				BP-				HK						HK					P										
Hydralazine																													
Nitrate			BP-																										
Loop diuretic	BP-				BP-	BP-	LK		LK	LK	LK								P										
Warfarin												B	B										B	B	B	B		P	B
Aspirin																					P	B	B	B	B				
Amlodipine	BP-																			P						P	M		

Key:

BP- = hypotensive effect; B=bleeding; HR-= bradycardia; VA = ventricular arrhythmias; LK/HK = effect on serum potassium; P= clinically significant altered plasma concentration which might require dosage alteration or closer monitoring; M=myopathy; CNS = central nervous system toxicity.
